# Faecal (or intestinal) microbiota transplant: a tool for repairing the gut microbiome

**DOI:** 10.1080/19490976.2024.2423026

**Published:** 2024-11-05

**Authors:** Rohma Ghani, Despoina Chrysostomou, Lauren A Roberts, Madhumitha Pandiaraja, Julian R. Marchesi, Benjamin H. Mullish

**Affiliations:** a Division of Digestive Diseases, Department of Metabolism, Digestion and Reproduction, Faculty of Medicine, Imperial College London, London, UK; b Department of Infectious Diseases, Hammersmith Hospital, Imperial College Healthcare NHS Trust, London, UK; c Department of Gastroenterology, St Mary’s Hospital, Imperial College Healthcare NHS Trust, London, UK; d Department of Hepatology, St Mary’s Hospital, Imperial College Healthcare NHS Trust, London, UK

**Keywords:** Faecal microbiota transplant, gut microbiome, metabolome, antibiotic resistance

## Abstract

Faecal/intestinal microbiota transplant (FMT/IMT) is an efficacious treatment option for recurrent *Clostridioides difficile* infection, which has prompted substantial interest in FMT’s potential role in the management of a much broader range of diseases associated with the gut microbiome. Despite its promise, the success rates of FMT in these other settings have been variable. This review critically evaluates the current evidence on the impact of clinical, biological, and procedural factors upon the therapeutic efficacy of FMT, and identifies areas that remain nebulous. Due to some of these factors, the optimal therapeutic approach remains unclear; for example, the preferred timing of FMT administration in a heavily antibiotic-exposed hematopoietic cell transplant recipient is not standardized, with arguments that can be made in alternate directions. We explore how these factors may impact upon more informed selection of donors, potential matching of donors to recipients, and aspects of clinical care of FMT recipients. This includes consideration of how gut microbiome composition and functionality may strategically inform donor selection criteria. Furthermore, we review how the most productive advances within the FMT space are those where clinical and translational outcomes are assessed together, and where this model has been used productively in recent years to better understand the contribution of the gut microbiome to human disease, and start the process toward development of more targeted microbiome therapeutics.

## 1. Introduction

The contributions of the gut microbiome toward the maintenance of homeostasis in humans and other mammals are well-recognized, including key roles in nutrient digestion and host metabolism, provision of ‘colonization resistance’ to protect against invading pathogens, and regulation of host immune system responses.^1,2^ The application of novel next generation sequencing and other ‘multi-omic’ techniques to patient samples – coupled with advances in bioinformatic tools to analyze such datasets – has enabled characterization of distinctive patterns of perturbation of the gut microbiome in a wide range of disease types, including those both within and beyond the gastrointestinal tract.^3^ Experimental approaches (using mouse models,^4^ approaches based *ex vivo* (e.g. bioreactors/chemostats,^5^ and other techniques) have enabled the transition from purely recognizing an association between a disease state and microbiome characteristics toward identification of specific mechanisms by which the microbiome contributes toward the pathogenesis of particular conditions.

Alongside this expanded knowledge of the role of the gut microbiome in health and disease, there has also been growing interest in approaches to restore the pre-morbid gut microbiome. A variety of different ‘microbiome therapeutic’ strategies exist and are currently widely employed in an attempt to restore the gut microbiome. These include prebiotic supplements, defined as non-digestible substances that can that alter the activity and composition of the gut microbiota to benefit host health, particularly (but not exclusively) via their fermentation. Alternatively, probiotics may be defined as live nonpathogenic microorganisms, which when ingested in sufficient amounts, can confer beneficial effects to the host. The beneficial effects of probiotics and prebiotics (as well as in combination) have been already explored in the context of a number of specific diseases states, both infectious as well as non-communicable (Supplementary Material for an overview of use in selected clinical scenarios). Although probiotic and prebiotic treatments therefore have a demonstrable role in contributing toward the restoration of the gut microbiome, the strategy to alter the gut microbiome with by far the greatest degree of apparent potential efficacy has been intestinal microbiota transplant (IMT, also known as ‘faecal microbiota transplant’ (FMT); our personal preference is use of the term ‘IMT’ – for reasons previously-outlined^6^ – although we predominantly use the term ‘FMT’ here based on convention and familiarity. The clearest clinical paradigm for its successful use is in the context of recurrent *Clostridioides difficile* infection (CDI)^7^ – a condition typically characterized by antibiotic-related microbiome perturbation – with FMT now recommended within the recurrent CDI treatment algorithm in several national guidelines.^5,8^ A key unique characteristic of FMT as a microbiome restoration strategy is its ‘whole microbiome/whole ecosystem’ approach, which may particularly account for its success in the role of restoring an antibiotic-damaged microbiome. Conversely, however, a key aim for the field is developing next-generation microbiome therapeutic medicinal products that build upon the foundational principles of FMT but seek to simplify and refine the approach. Examples of this include the FDA-approved spore-based therapeutic Vowst/live-brpk (licensed within the United States for the treatment of recurrent CDI)^9^ and the promise of a defined bacterial consortium clinically for the same indication.^10^

However, FMT is a not a panacea. While systematic review/meta-analysis demonstrates that FMT is much more efficacious in treating recurrent CDI than antibiotics alone,^11,12^ the number of patients requiring more than one FMT (or related live biotherapeutic products) to gain remission – or failing to gain remission despite a course of FMTs – is small, but not trivial, with the understanding of factors that may influence treatment failure being only partially understood.^13,14^ Similarly, while FMT has shown promise in randomized trials for treatment of a wide range of non-CDI conditions (with the full range of non-CDI conditions in which it has been trialed having been recently reviewed),^15^ it has overall shown much lower success compared to CDI, and as such is not currently recommended in guidelines for routine use in any other medical indication.^13,14^

**Figure 1.**
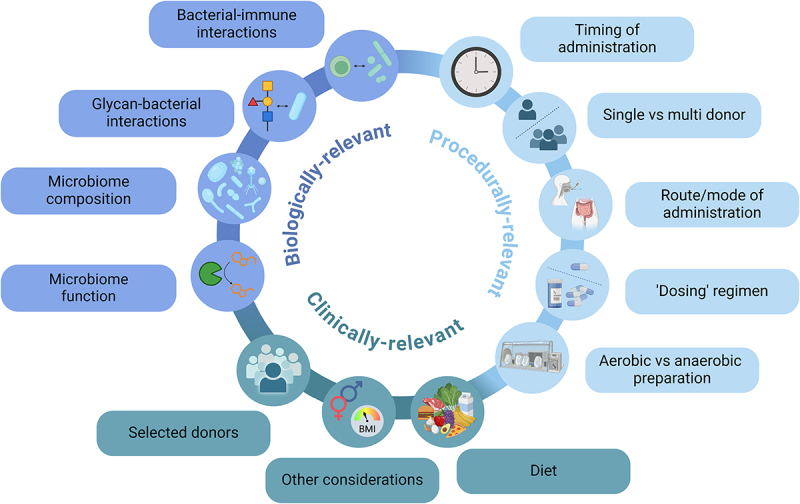
Summary of key clinical, biological, and procedural factors, which may influence the efficacy of FMT in repairing the gut microbiome and/or as a therapeutic tool.

In this review, we investigate clinical, biological, and procedural factors that may influence the efficacy of FMT in repairing the gut microbiome (these are summarized in Figure 1). A better understanding of the existing evidence in the field, and current gaps in knowledge, may help to optimize selection of donors, recipients, or donor-recipient matching, and guide the development of ‘next generation/post-FMT’ targeted microbiome therapeutics. Throughout, we have tried to emphasize how findings from clinical FMT studies have helped to inform translational science studies, as well as how knowledge from fundamental scientific investigation has been adopted into FMT studies.

## 2. Factors related to success of FMT in repairing the gut microbiome

### 2.1. Clinically relevant factors

#### 2.1.1. General considerations

The evidence that exists for key basic clinical aspects of donor selection (including related *vs* unrelated, donor age, and donor sex) suggests that such factors overall do not greatly influence the efficacy of FMT in resulting in a positive clinical outcome, at least in the context of recurrent CDI (where the body of evidence is largest).^13^ However, recent evidence suggests the matching of these factors between donor and recipient may be important – specifically, a sex-discordant FMT between donor and a recipient with recurrent CDI was associated with an increased risk of subsequent irritable bowel syndrome (IBS; particularly of mixed type) in both univariate and multivariate analyses.^16^ No clear hypothesis has been currently proposed that could clearly explain these findings.

Originally, there were concerns that particular chronic comorbidities of recipients would adversely affect the efficacy of FMT. Overall, while the evidence regarding the impact of specific comorbidities on the efficacy of FMT is weaker for certain comorbidities^13^ and stronger for certain others,^17^ most individual clinical studies have not shown any comorbidity that consistently correlates with a significant increase or decrease in FMT efficacy. Such comorbidities that do not appear to affect efficacy (at least in the context of CDI) include cancer, a range of forms of immunocompromise/immunosuppression, solid organ transplant, chronic liver disease, cardiovascular disease, diabetes mellitus, recurrent urinary tract infection, and COVID-19 infection.^13^ Patients with CDI with underlying IBD appear to overall have comparably good outcomes from the CDI perspective compared to those without IBD, with the risk of IBD flare post-FMT appearing lower in prospective studies than was originally suggested by retrospective studies.^18,19^ Again principally in the context of recurrent CDI, there are very few individual studies that have demonstrated that demographic factors (including age, sex, and body mass index (BMI) of recipients), and infection-related factors (including number of prior CDI episodes, strain of pathogen, and hospitalization due to CDI) that closely influence efficacy, with prior medication use (including proton pump inhibitors (PPIs), corticosteroids, lactulose, or probiotics) also not obviously associating with outcome.^13^ However, a meta-analysis of 20 studies, containing data from 4,327 people treated for recurrent CDI with FMT, demonstrated that severe CDI, prior CDI-related hospitalization, inpatient status, and quality of bowel preparation were significant predictors for FMT failure; conversely, Charlson Comorbidity Index, female gender, immunosuppressed status, and number of prior CDI recurrences were not associated with failure.^20^ The publication of data from certain large FMT registry studies have also suggested factors that may linked to outcome; analysis of 658 FMTs performed for CDI in France in 617 patients from 2018 to 2022 suggested certain further factors associated with FMT failure, including severe chronic kidney disease, partial retention of FMT, and (as per the described meta-analysis) insufficient bowel cleansing.^21^

Certain studies have attempted to integrate clinical and other relevant variables to optimally select donors or better delineate clinical factors associated with FMT success. A high-throughput FMT center in Denmark used a mixed-effect model analysis to demonstrate that patient age >65 years, non-CDI antibiotics at week 1 post-FMT, and the donor used were all associated with the degree of efficacy of FMT. However, the analysis found that neither increasing the dose of fecal microbes nor standardizing processing protocols improved outcomes.^22^

The data outlined here evidently focus particularly on CDI, given that this is the disease with the largest and strongest evidence base for FMT’s use. However, it is clearly reasonable to hypothesize that donor and recipient clinical factors associated with optimal outcome from FMT may be disease specific, and factors that appear of limited importance to consider in the context of CDI may be of greater pertinence in non-CDI conditions. However, there is not clearly any such consistent signal in any non-CDI setting. For instance, a recent Delphi study evaluating FMT in IBD (the most intensively studied non-CDI condition in the context of FMT) considered all the factors mentioned above, together with more IBD-specific clinical factors (degree of activity/inflammation, level of immunosuppression), but could not currently make any conclusions regarding whether any such factors influenced outcome from FMT, and how they should influence future trial design.^23^

#### 2.1.2. Highly selected donors

Certain studies have explored selecting an optimal donor based on clinical characteristics more generally associated with a ‘beneficial microbiome’. However, this approach is extremely challenging due to the marked inter-individual variability of a microbiome, the temporal dynamics within an individual of their microbiome, and the lack of definition of what constitutes a ‘healthy microbiome’.^24^ In one high-profile example, a Norwegian group selected a stool donor for an IBS/FMT study based upon them being a young, athletic, nonsmoker, taking no medications, with a low BMI, vaginally delivered and breast-fed, with minimal antibiotic exposure, and with a healthy diet;^25^ it is not clear how many donors were screened in the process. While use of this ‘superdonor’s’ stool was associated with marked improvements in IBS symptoms in this particular study, meta-analysis of FMT/IBS trials is overall negative or of modest benefit, with other procedurally similar FMT/IBS trials to the ‘superdonor’ study also not reproducing similar clinical benefits.^26^

A number of other FMT studies have tried a similar approach to selecting healthy donors but have not been able to clearly demonstrate an impact of a highly-selected donor on clinical outcomes. As a comparable strategy, other studies have tried to select donors based on a clinically desirable phenotype, sometimes an ‘extreme phenotype’, assuming that this feature is microbiome-related in that donor, e.g. low BMI donor despite high calorie intake in study of FMT for obesity.^27^ In a double-blind pilot RCT investigating the effect of FMT from a lean donor in obese patients, although there were sustained changes in the microbiome profile of obese patients, there was no clinically significant impact on lowering BMI.^27^ Conversely, two clinical trials evaluating the safety and efficacy of FMT from stool donors with preserved response to anti-PD-1 immune checkpoint inhibitors showed promising results in overcoming resistance to anti-PD-1 immunotherapy.^28,29^ However, these studies had no comparator arm using ‘standard’ healthy donors, making it difficult to ascertain if the response seen was related to using a drug responder, or just a general effect. A further practical difficulty of using ‘responding patients’ more generally as stool donors is the question as to whether they will pass other aspects of conventional FMT donor screening criteria, that will enable their stool to be processed within a regulated FMT lab using standard protocols. Furthermore, caution is required when selecting donors of extreme phenotypes, as there is a risk of transmission of adverse (and not just beneficial) phenotypes. For example, in a clinical study investigating the effect of donor phenotype on glucose metabolism and intestinal homeostasis, patients with metabolic syndrome receiving FMT from a donor with metabolic syndrome experienced a transient significant decrease in insulin sensitivity at 2 weeks post FMT, an effect that was not observed in patients where the donor had undergone Roux-en-Y gastric bypass (RYGB) bariatric surgery.^30^ Such scenarios highlight the importance of striking a fine balance between reaping the full potential benefit of FMT donor selection and exercising caution with the risk of transmitting undesirable, adverse effects.

#### 2.1.3. Diet

Patients and clinicians alike have directed curiosity toward the role of donor and recipient diets in influencing FMT outcomes. A questionnaire-based study of healthcare professionals with interest and expertise in FMT found that there was a real demand for evidence-based advice on this topic;^31^ however, currently there is limited overall evidence, including a lack of even general advice in FMT guidelines.

Prebiotics have been one particular area of interest, given their fermentability by the gut microbiome. A small pilot study suggested increased fiber in the diet of FMT recipients may result in improved success of FMT in CDI and ulcerative colitis (UC).^32^ However, a further phase II randomized clinical trial randomized people with metabolic syndrome to receive oral FMT together with either high or low fermentable fiber supplementation. The expectation was that the high fermentable fiber group may see particular benefit, as it was predicted that this fiber would be fermented into metabolites such as short chain fatty acids (SCFAs), which have been associated with a number of host health benefits including related to reduced insulin resistance.^33^ However, the study had an unexpected finding of improvement at 6 weeks in insulin resistance in those receiving low fermentable fiber, but not in those receiving high fermentable fiber.^34^ This benefit may have been related to the low fermentable fiber’s impact upon gut motility, which itself may in turn impact the gut microbiome; low fermentable fiber use has also been associated with altered host immune responses, reduced vulnerability to infection, and other impacts upon host physiology.^35^ Meat-eating status may also be important, with a group in the Netherlands exploring the impact of using lean vegan donors where FMT is being used to treat metabolic conditions, based upon the distinctive microbiome composition and pattern of carnitine metabolism associated with meat eaters compared to vegans.^36^ The utility of this strategy in FMT has mixed supporting evidence, with one human trial showing beneficial effects of FMT derived from lean vegan donors as compared to autologous FMT on markers of hepatic steatosis,^37^ whereas another RCT demonstrated no appreciable impact on inflammatory parameters among individuals with metabolic syndrome.^38^ A third area of focus has related to the use of the Mediterranean diet, with somewhat complex results. A small pilot RCT by Koopen and colleagues reported that the combination of FMT and Mediterranean diet in people with metabolic syndrome provided no additive benefit compared to just receipt of a Mediterranean diet alone.^39^ However, in a further study, obese or dyslipidemic patients were first assigned to receive either general dietary advice, a Mediterranean diet alone, or a ‘green-Mediterranean’ diet (containing green tea and *Wolffia globosa* (also known as Asian watermeal or duckweed) green shakes); all patients also received free gym membership and physical activity guidelines.^40^ At 6 months, participating patients provided stool samples and received either autologous FMT or placebo between months 6 and 14. Only the group receiving autologous FMT after the green Mediterranean diet had reduced weight regain (and other minimally changed metabolic parameters) over the follow-up period. Micronutrients and FMT outcome have been modestly explored only, but with some findings of potential interest. In particular, after FMT for recurrent CDI, a small study suggested that zinc deficiency appeared to predict recurrence, with apparent reduced recurrence risk in those receiving zinc supplementation.^41^

Collectively, while these data suggest an impact of dietary manipulation upon FMT outcome, they are not clear-cut and straightforward to interpret. With greater insight into the mechanistic basis for the influence of dietary factors on outcome parameters in FMT studies, we may be able to harness this factor to maximize favorable outcomes in the growing field of FMT therapeutics.

### 2.2. Biologically relevant factors

#### 2.2.1. Gut microbiome composition and functionality

Several recent meta-analyses of paired donor, pre-FMT and post-FMT metagenomes of FMT performed for a wide range of indications have given more granular insight into the shift in microbiome dynamics associated with FMT.^42–44^ One of these studies observed that the higher the degree of engraftment (i.e. donor bacteria that had transferred into and become incorporated into the recipient microbiome), the more likely clinical success was.^42^ The same study also concluded that FMT from different routes, and with FMT pre-treatment with antibiotics (where FMT was being used to treat an infectious disease) was associated with higher engraftment. However, an alternative study suggested that the relationship between engraftment and treatment success only applied to particular indications,^43^ while a further comparable study did not find a clear relationship between engraftment and outcome at all.^44^ One of these studies noted that species from within the bacterial phyla Bacteroidetes and Actinobacteria species overall displayed higher engraftment compared to Firmicutes,^42^ while no specific colonizing or persisting bacteria were identified in another study.^44^ This variability in outcome may be potentially reflective of the different bioinformatic strategies used in analysis; all of these meta-analyses recognized the wide heterogeneity in engraftment between different FMT trials. It has also been difficult to discern whether the potential association between antibiotic pre-treatment and successful FMT can be attributed to the use of antibiotics *per se* or was confounded by the fact that the majority of patients being treated with prior antibiotics had an infection, i.e. CDI, or being treated because of intestinal carriage of multidrug-resistant bacteria.^42^

Ecological metrics are also of interest and relevance. A consistent finding throughout studies of FMT in recurrent CDI has been that gut microbiome alpha diversity increases significantly and durably after FMT, reaching levels comparable to healthy donors. A number of FMT studies in non-CDI settings have demonstrated an increase in alpha diversity in association with FMT use, with data in certain disease types suggesting that increased alpha diversity of a donor associates with success, e.g. in a systematic review of 25 FMT/UC studies.^45^ Comparably to this, another study compared microbiota composition between two donors used in studies of FMT for UC (one with apparent high efficacy, one with much lower efficacy) and concluded that donor microbiota stability and evenness associated with success of FMT.^46^ One of the above-described meta-analyses concluded that alpha diversity associated with engraftment, but did not impact upon the success of FMT.^43^

As such, these studies do not present easy answers to allow sequencing-based selection of an ideal donor. To advance this, novel bioinformatic tools may offer one approach – within the FMT literature, these have evolved over recent years, from the original use of simple machine learning approaches to select optimal donors from a stool bank based on levels of ‘beneficial families’ within a stool donor microbiome,^47^ to more sophisticated machine learning approaches to predict FMT outcome, including application of random forest models and logistic LASSO regression.^42,44^ However, as of yet, there is no published study that has convincingly demonstrated use of machine learning approaches to improve donor selection and enhance efficacy of FMT in a clinical context.

An alternative proposed strategy may be to use existing pre-clinical and clinical data – both observational and experimental – to attempt to rationally select out donors of interest based upon gut microbiome characteristics. As an example – one area of growing interest has been the use of FMT as a tool to diminish the sequelae related to intestinal colonization with multidrug-resistant organisms (MDROs), either by reducing their load within the gut/decolonization, and/or mitigation of invasive infection related to them.^6,48^ ‘Colonization resistance’ is the term used to describe the way in which the microbiome operates both directly and indirectly to prevent colonization and invasive infection from pathogens, as well as to provide immune regulation. Table 1 lays out a range of experimental mouse and human studies that have identified a selection of commensal bacteria associated with potential colonization resistance against MDRO. This raises the question of whether selection of donors whose gut microbiome is ‘enriched’ in particular commensals on this list (particularly some of those more consistently mentioned, including *Bifidobacteria, Faecalibacterium prausnitzii*, or *Blautia producta*) would be considered ideal donors for FMT particularly used for patients with intestinal MDRO carriage. If so, is this approach based on (a) the presence of the bacteria in stool detected by metataxonomic and/or metagenomic approaches, (b) by a threshold of number of viable bacteria (e.g. measured by colony forming units) within a particular stool donation or stool donations over time, (c) checking for some functional aspect of these commensal bacteria? Regarding (c) – in particular, *Blautia producta* has been described as inhibiting the growth of vancomycin-resistant Enterococci via production of a lantibiotic that exerts a bacteriostatic effect,^70^ while other commensals appear to restore colonization resistance and protect gut barrier integrity through production of metabolites including bile acids and SCFAs; these functions could potentially be tested for. Even if such commensals are present within the fresh donated stool, it remains unknown if they will be retained during FMT processing to still be viable within the final therapeutic product administered to patients. This strategy is theoretically attractive but remains untested.

**Table 1.** Commensal bacteria associated with limitation of intestinal pathobiont colonization and infection

Reference	Commensal	Type of study	Summary
49	*Prevotella stercorea*	Human	These taxa all elevated in the gut of liver transplant patients who were never colonized with an MDRO
	*Bacteroides*		
	*Bifidobacterium*		
	*Faecalibacterium prausnitzii*		
	*Parabacteroides distasonis*		
	*Prevotella copri*		
50	Bacteroidales	Human	Associated with absence of MDRO colonization
51	*Akkermansia muciniphila*	Human	Gut levels significantly greater in subjects who did not acquire an MDRO
	*Odoribacter laneus*		
52	*Bacteroides*	Human	Decreased in the gut of critically ill patients colonized with CRE
	*Barnesiella*		
53	*Bifidobacterium bifidum*	Human	Significantly increased post-FMT in “responders”
54	*Bacteroides*	Human	Higher proportion in donor stool more likely to be associated with a response to FMT
	*Barnesiella*		
	*Butyricimonas*		
55	Atopobiaceae	Human	Higher abundance in gut microbiome nursing home residents never colonized with an MDRO *versus* those colonized
	*Dorea*		
	Lachnospiraceae ND3007		
56	Bacillales Family XI incertae sedis	Human	Presence of these bacteria in gut microbiome associated with lower risk of colonization by MDRO, infection, and death
	*Prevotella* spp		
57	*Coprococcus*	Human	Significantly more abundant in the gut microbiota of individuals not colonized with ESBL-E
	*Desulfovibrio*		
	*Oscillospira*		
	*Parabacteroides*		
58	*Prevotella massiliensis*	Human	Higher abundance in the gut microbiome of healthy adults who were not colonized with an MDRO *versus* those colonized
	Pseudomonadaceae		
59	*Bacteroides dorei*	Human	Presence of these bacteria within gut microbiome associated with CPE negative stool samples
	*Faecalibacterium prausnitzii*		
	*Bifidobacterium pseudocatenulatum*		
	*Bifidobacterium bifidum*		
	*Colinsella aerofaciens*		
	*Eubacterium rectale*		
	*Streptococcus salivarius*		
60	Ruminococcaceae	Human	Presence of these bacteria associated with a decreased risk of intestinal Gram-negative microbiome domination
61	*Blautia producta*	Human	Rectal colonization with VRE was inversely associated with presence of *B. producta*
62	*Lactobacillus* spp	Mice	Patients who did not colonize with an MDRO were more likely to be colonized with this
63	*Blautia producta*	Mice	Prevented colonization and had antimicrobial activity against *Listeria monocytogenes*
	*Hungatella hathewayi*		
	*Thomasclavelia ramosa*		
	*Thomasclavelia saccharogumia*		
64	*Blautia producta*	Mice	Commensals associated with clearance of VRE colonization
	*Clostridium bolteae*		
65	*Ruminococcus gnavus E1*	Mice	*Clostridium perfringens* was eliminated from the digestive tract by this strain
66	*Lactobacillus paracasei* CNCM I-3689	Mice	Presence of this associated with significantly decreased VRE burden in the feces
67	*Bacteroides thetaiotaomicron*	Mice	Repressed Shiga toxin 2 mRNA expression (virulence factor in *E. coli* O157:H7)
68	*Ruminococcus obeum*	Mice	Restricted *Vibrio cholerae* colonization
69	*Lactobacillus* spp	Mice	Controlled *E. coli* multiplication in small intestine and stomach
70	*Blautia producta* BP_SCSK_	Mice	Inhibited colonization of VRE in intestine
71	*Lactobacillus* HT121	Mice	Administration reduced intestinal VRE burden
	*Lactobacillus* Y74		
72	*E. coli* HS	Mice	Prevented colonization with *E. coli* O157:H7
	*Escherichia coli* Nissle 1917		
73	*Bifidobacterium animalis* subsp. *lactis*	Mice	Reduced the fecal load of *Salmonella* in gnotobiotic mice
74	*Klebsiella oxytoca*	Mice	Inhibited intestinal *Klebsiella pneumoniae* colonization
75	*Bacteroides thetaiotaomicron*	Mice	When administered to mice they were less likely be colonized with VRE
76	*Barnesiella*	Mice	Reconstitution with *Barnesiella* correlates with VRE elimination
77	*Bifidobacterium animalis*	*In vitro*	Antimicrobial activity against *Staphylococcus aureus, E. coli*, and *Pseudomonas aeruginosa*
	*Bifidobacterium bifidum*		
	*Lactobacillus casei*		

**Figure 2.**
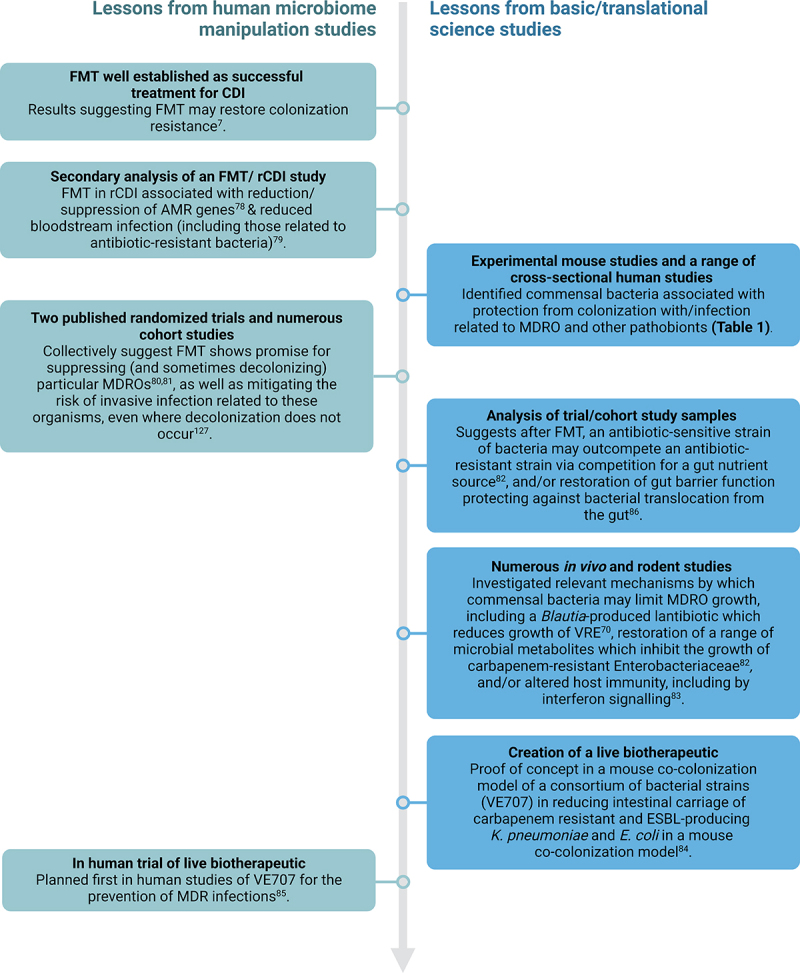
Interplay between human FMT and fundamental/translational science studies in advancing understanding of gut microbiome to human disease and in microbiome therapeutics for it. In this case, using the scenario of antimicrobial resistance/multidrug-resistant organisms as an example. Created in BioRender.com.

CRE: carbapenem-resistant Enterobacterales; ESBL: extended-spectrum beta-lactamase-producing Enterobacterales; VRE: vancomycin-producing Enterococci. Certain other key studies^78–86^ are discussed in more detail in the text and in Figure 2.

A further related approach of interest may be to consider non-bacterial aspects of the gut microbiome and how they may relate to FMT’s efficacy. Although FMT efficacy has been conventionally attributed to the presence of live bacterial cells in the final FMT product, certain studies have appeared to demonstrate that sterile FMT– with live bacteria removed through filtration – may be similarly effective to conventional FMT. The provocative demonstration in five patients with recurrent CDI that sterile filtered FMT appeared of comparable efficacy to conventional FMT has been one of the launchpads for research focused around the search for soluble mediators underlying the efficacy of FMT^87^ (of note, in a recent randomized trial of lyophilized fecal filtrate *versus* lyophilized FMT in treating recurrent CDI that has been presented in abstract form, conventional FMT resulted in a much higher cure rate than filtrate, but – in the absence of a placebo/control arm – it is unknown if the filtrate itself resulted in any higher rate of cure than might have been expected for conventional anti-CDI antibiotics alone.^88^ Considering such possible mediators, the gut virome might contribute to the pathogenesis of CDI and efficacy of FMT; for instance, CDI patients receiving FMT have shown beneficial clinical outcomes associated with an increase in donor-derived Caudovirales. Bacteriophage profiles have been associated with success of FMT for rCDI in other studies performed, characterized by an apparently closely related decrease in the abundance of Proteobacteria with increase in Microviridae.^89,90^ Outside of the CDI setting, fecal virome transplant alone has been demonstrated to improve obesity and glycemia in mice.^91^ However, once again, no study has meaningfully used phage profiling to improve donor selection and enhance clinical outcomes. As an alternative approach – a number of studies have demonstrated that particular gut microbial metabolites may underlie the efficacy of FMT in particular scenarios. For example, in the context of CDI, FMT-related restoration of SCFAs (and particularly the five carbon variant, valerate) – together with restoration of bacteria with bile salt hydrolases (BSH) and other bile-metabolizing enzymes that restore the pre-morbid gut bile acid *milieu* – results in reduced triggers to *C. difficile* germination and impaired growth and toxin production by the organism.^92–95^ These data may lead one to select donors with high gut BSH functionality and may be a useful screening test in donor selection for rCDI. In a similar vein, metabolomic screening of stool from potential FMT donors may also be an approach of interest, e.g. to look for high ‘valerate producers’. However, one practical issue with this approach is that metabolomic assays are often cumbersome to perform, with (in the case of mass spectrometry) batch-to-batch variability in quantification; furthermore, levels of SCFA in stool may vary considerably depending upon recent diet and time of stool production,^96^ meaning that simple binary selection of, for example, ‘high and low SCFA producers’ may not be straightforward.

Conceptually, there are further rational approaches that might be employed to select donors or match donor and recipient based upon gut microbiome functionality. One such approach relies on considering a biological target that it is hoped the FMT might act upon. As an example – if aiming to treat IBS with FMT, given that high gut methane production is associated with reduced gut motility and worsened IBS with constipation, would it be rational to select out recipients likely to benefit based upon methane production, and select out donors based upon methane-degrading functionality of their gut bacteria? A more sophisticated approach may be to combine a variety of functional type assays to refine donor selection further. In a recent study of FMT in patients with UC, 12 donors were selected on a combination of stool microbiota composition, the ability of stool fraction libraries to induce both regulatory T cell (T_reg_) production in the colonic lamina propria and SCFA production in the cecum of germ-free mice.^97^ The study reported that one particular selected donor’s stool appeared particularly effective, despite apparent low donor microbiome engraftment.

#### 2.2.2. Other potentially relevant biological factors

There are a number of other feasible biological factors that may be relevant to FMT that have not been well-explored. As one example, the case is made for further consideration of donor and recipient genetics.

Human genetic variation is well-established to influence the gut microbiota. In one study of 5,959 genotyped patients with matched gut microbial metagenomes, 567 independent SNP–taxon associations were identified. The significance of some associations varied based upon the presence or absence of particular dietary components, some were related to genes involved in immune and metabolic processes, while certain bacterial taxa associated with genetic loci associated with specific disease types (including colorectal cancer).^98^ Rodent studies have already identified that mutations in specific genetic loci are altered with an altered gut microbiota, with one example being *Nod2* knockout mice with increased susceptibility to colitis-associated colonic cancer; this phenotype is transferable by microbiome transfer.^99,100^ As such, one possible extrapolation that might be made from such data is that the genetics of the donor, through an influence upon the gut microbiome, is a change toward a gut microbiome that is able to transmit disease traits. This raises the possibility of a potential future role for targeted genetic screening for the optimal selection of donors, with the aim of attempting to screen out donors with potentially ‘disease transmitting’ functionality.

A further observation in the genotype–metagenome association study highlighted above was an association between levels of the bacterium *Faecalicatena lactaris* and the *ABO* blood group, consistent with other research exploring the association between particular bacteria and secreted blood antigens as an energy source within the gut.^98^ The *ABO* blood group is of interest, as this is clearly an important paradigm for the matching of donated blood between donors and recipients, and a key factor to minimize risk of rejection in solid organ transplants. Recent evidence has also demonstrated that the presence rate of a structural variation segment in *Faecalibacterium prausnitzii* that harbors an *N*-acetylgalactosamine (GalNAc) utilization gene cluster is higher in people who secrete the type A oligosaccharide antigen terminating in GalNAc, a feature that is jointly determined by human *ABO*;^101^ the same study also demonstrated that other *ABO*-associated species apart from *F. prausnitzii* can also utilize GalNAc, particularly *Collinsella aerofaciens*. Furthermore, the degree of binding of *C. difficile* toxins (both TcdA and TcdB) appears to be related to blood group, with more potent binding to blood group A oligosaccharide compared to blood group B.^102^ It would be pertinent for future studies to explore the relationship between blood group in donors and recipients and FMT outcome. For instance, donor–recipient *ABO* concordance may be favorable to facilitate colonization of bacteria from the donor that has already adapted to the glycan environment of the host; furthermore, patients with blood group A may be harder to treat with FMT for CDI in light of stronger *C. difficile* toxin binding.

### 2.3. Procedurally relevant factors

#### 2.3.1. General considerations

The current evidence base is that a number of much-debated preparation-related factors related to laboratory handling of FMT (including temperature of freezer storage, use of lyophilization, type of capsule used, processing time, and emulsion process) appears to minimally impact upon the efficacy of FMT, at least in the context of CDI.^13^ However, this evidence is mostly weak quality, and whether these findings can be extrapolated from CDI to other disease processes is uncertain. A controversial topic is the relevance of preparing FMT within an anaerobic environment; this would intuitively make sense, given the dominance of anaerobes within the gut environment. One *ex vivo* study suggested that a full anaerobic environment was not necessary, and removal of air above collected samples was sufficient for preservation of bacterial viability.^103^ Conversely, other studies have suggested that anaerobic processing may be useful to preserve viability and microbiome functionality.^104,105^ A practical difficulty in performing and interpreting the applicability of *in vitro* and *ex vivo* studies in this area is selection of an appropriate outcome measure. As microbiome sequencing would not be able to differentiate dead from live bacteria, it remains unclear if overall bacterial viability, viability of particular taxa, or quantifying some element of microbiome functionality would be most appropriate. At present, there is no consistently clear signal characterizing the impact of anaerobic preparation on FMT efficacy in any disease group.

A further area of discussion relates to the use of antibiotics and/or bowel purgatives prior to FMT. There are several intuitive reasons why this may be beneficial, including creation of a microbiome/ nutritional/ metabolic niche into which the FMT may colonize, or purging of any residual pathogenic bacteria or antibiotic remnants (the latter of potential relevance particularly in the case of CDI). There is at least a degree of evidence supporting use of these agents from microbiome analysis within FMT studies, as outlined above. Evidence from rodent studies and human trials for the role of these agents has been recently reviewed;^100^ the evidence for FMT colonization after antibiotics appears overall stronger in mice than in human FMT studies, which may be reflective of the tendency to use supratherapeutic antibiotic doses within rodent studies leading to a more disrupted gut microbiome. This is a further area where there is no clear current clinical consensus to guide best practice.

#### 2.3.2. Route/mode of delivery

Aspects related to route and mode of delivery of FMT and its potential impact on efficacy have also been debated. Potential routes of administration of FMT include the following: nasoenteral tube (often nasojejunal, but also nasogastric), endoscopic (particularly via colonoscopy, but with other options including gastroscopy or flexible sigmoidoscopy), retention enema, or capsulized administration.^106^ A small number of studies have employed combined administration routes, including simultaneous bidirectional FMT.^107^ Certain considerations of the route chosen relate to patient factors (e.g. frail elderly patient may not be able to retain enema or may have poor acceptance of an unacceptable risk from colonoscopy), but the evidence base for success associated with different routes is clearly a major consideration for that which is chosen.

In CDI, there appears to be a small advantage to a colonoscopic route of administration compared to other routes of administration,^12–14^ and a clear case for a high likelihood of remission from second or further FMTs if a first FMT has not been successful. However, optimal route of administration may be a disease-specific characteristic – for example, a recent meta-analysis of seven randomized controlled trials of FMT use in patients with irritable bowel syndrome (IBS) demonstrated that FMT was superior to placebo in improving IBS symptoms when administered either via lower or upper gastrointestinal endoscopy (RR 0.70, 95% CI 0.51–0.96; RR 0.37, 95% CI 0.14–0.99, respectively), but FMT was inferior to placebo in IBS symptom relief when administered as oral capsules (RR 1.88, 95% CI 1.06–3.35).^108^ While capsulized FMT is of clear preference to patients compared to invasive administration routes such as colonoscopy, it is important to recognize that capsulized preparations may not be advantageous in all scenarios; whether this reflects an intrinsic aspect of the capsulized design (‘dose’ of material, impact of lyophilization, etc.) – or a factor more related to upper GI administration of fecal bacteria as opposed to lower GI administration – will require further research to determine.

#### 2.3.3. Dosing of FMT

‘Dosing’ of FMT is a difficult concept. While certain studies have made inferences about weight of stool per FMT and therapeutic success,^109,110^ weight of stool is not a marker of any biological characteristic such as bacterial load, and its use may be limited. While one FMT alone is sufficient for most cases of recurrent CDI, it is clear that there is at least some element of ‘die off’ of effect – based upon clinical assessment, or microbiome profiling – when FMT is used in non-CDI settings (spanning disease indications from UC,^111^ metabolic disorders,^37,112^ and chronic liver disease^113^), generally quoted as between a few weeks to a few months. In response to this, certain recent FMT study designs have shifted from a conventional course of a single or small number of serial ‘full’ FMTs toward more sustained ‘lower dose’ capsulized FMT.^114^ Recognizing an ongoing knowledge gap within this area, there has been a proposal for FMT and other microbiome therapeutics to be considered more within a pharmacological framework, with more focus upon formulation, pharmacodynamics, and pharmacokinetics.^115^

#### 2.3.3. Timing of FMT

Recent research has focused on the relevance of timing of FMT. Once again, the best understood paradigm is recurrent CDI. While FMT was initially viewed as a ‘last resort’ therapy for recurrent CDI primarily due to familiarity with and related to the safety of FMT, there has been a shift in when FMT should be introduced in the CDI pathway for successful clinical management of the disease. Recommendations indicate the FMT should be introduced earlier in the recurrent CDI disease pathway.^13,14^ In addition, this has been accompanied by recent trial data, suggesting that FMT (after anti-CDI antibiotics) during a first or second episode of CDI appears to be of comparable efficacy and safety compared to later within the disease course.^116,117^ This success of FMT early in the CDI disease course is to some degree intuitive – patients early in the CDI course most at risk of future recurrence have the most disrupted gut microbiome and metabolome, with conventional CDI treatments (including vancomycin) killing *C. difficile* but with the collateral damage of further microbiome disruption.^118–120^ As such, a microbiome therapeutic might be seen to have the greatest ‘value added’ early in the CDI disease course, for those at greatest risk of recurrence.

However, this apparent importance of timing of FMT might partly relate not just to the degree of microbiome disruption but also to the level of gut inflammation. Both antibiotic exposure and intestinal inflammation create niches that favor the expansion of Enterobacteriaceae within the gut^99^ but which are much less favorable to colonization of commensal obligate anaerobes from within the Bacteroidia and Clostridia phyla. This may have implications for the timing of FMT in gut inflammatory conditions, and particularly IBD. In a systematic review of FMT use in UC, less severe and extensive disease, and potentially shorter duration (<1 year) were seen as predictors of FMT response.^45^ Given early work from IBD inception cohorts has suggested that gut microbiome profiles at diagnosis may predict future trajectories,^121^ this may suggest that a role for FMT in IBD is early after diagnosis, in those with particular microbiome profiles and/or lower degrees of inflammation. However, use of FMT as an early or even initial treatment in IBD may be seen as controversial, given that FMT has shown signals of promise for induction comparable to (but not clearly greater than) conventional pharmacological agents but does not have an established clinical role within IBD and clearly has potential risks associated with it use.^23^ A further consideration for the use of FMT in patients with established IBD may be that its ideal role is potentially in consolidating remission after use of conventional potent anti-inflammatories to induce remission; using this rationale, one of the only randomized trials of FMT in Crohn’s disease administered FMT to patients receiving corticosteroids during a flare.^122^

Other contexts where the timing of FMT remains debated relates to scenarios where the use of FMT is in patients at risk of antibiotic exposure and/or related factors that may disrupt their gut microbiome. One such scenario is in the setting of hematopoietic cell transplantation (HCT) for hematological malignancy. These patients have altered gut barrier dysfunction, loss of microbiome diversity, and enrichment of gut pathobiont bacteria, partly related to the direct immunosuppressive effects of the disease and treatments used for it, and partly related to their predisposition to infections and the resulting frequent courses of antibiotics used to treat them.^60,123,124^ Large observational studies have demonstrated that low gut microbiome diversity prior to HCT associates strongly with poor post-HCT outcome, including graft-versus-host disease and risk of future mortality; expansion of *Enterococcus* across the peri-HCT period has also been linked to poor outcomes.^125,126^ It has been demonstrated that patient stool collected prior to HCT can be transplanted back to them after HCT cell engraftment safely, with reestablishment of pre-morbid diversity and composition.^124^ Our group and others have also used FMT and other microbiome therapeutics successfully in this setting,^54,127–131^ although debate persists about the optimal timing relative to HCT; this is discussed further in Table 2. While a pragmatic solution in those setting up trials in this space might be to consider combined pre- and post-HCT administration, this is resource-intensive and does not allow delineation of whether the pre-FMT or post-FMT strategy may biologically and clinically be of greatest benefit.

**Table 2.** Potential advantages and disadvantages to different timing strategies of FMT. In this case, focusing upon hematopoietic cell transplant as a particular example.

	FMT pre-HCT	FMT post-HCT
Advantages	The existing evidence base strongly links gut microbiome diversity *pre-* (rather than *post-*) HCT with post-HCT outcome,^125,126^ as well as observing the peri-HCT bloom in *Enterococcus* that is associated with poor future clinical outcomes. Collectively, this supports ‘prehabilitating’ the gut microbiome prior to HCT, aiming to minimize the opportunity for peri-HCT microbiome disruption. Similarly, early antibiotic use in the peri-allogeneic HCT period (and particularly use of antibiotics prior to HCT) is associated with lower urinary 3-indoxyl sulfate, reduction in particular commensal taxa, and higher transplant-related mortality than patients receiving antibiotics later.^132^ This again may support a strategy of targeting microbiome manipulation before the insult of antibiotics.	The very nature of HCT (and high likelihood of antibiotics in the peri-HCT period) means that any pre-HCT FMT will inevitably be at least partly disrupted by these factors, mitigating its effect upon diversity/taxonomy. Administering FMT in a period of clinical stability post-HCT means directly boosting the gut microbiome at its lowest nadir of diversity and most disrupted, hopefully where there are minimal insults ahead that may kill off bacteria from within the FMT. Based upon data presented elsewhere in this paper, engraftment may potentially be greatest post-HCT, given the level of microbiome disruption at this point (i.e. very low alpha diversity).
Disadvantages	FMT pre-HCT will mean additional hospital visits by the patient to a Haematology Centre, adding further burden to an already intense patient visit schedule pre-HCT. Scheduling will need to be very carefully timed, balanced between avoiding the cell nadir related to prior chemotherapy, and long enough prior to HCT to enable successful engraftment and recovery. Any side effects occurring in relation to the FMT (e.g. infection, side effects related to an invasive administration protocol) may require further treatment. This may slow progression toward receiving HCT, and potentially negatively impact upon the outcome of the hematological malignancy.	Clinical studies to date have typically waited until after neutrophil engraftment before administering FMT to mitigate the risk of infective complications/further microbiome insults – but this may have meant missing a window within which the FMT itself may have influenced clinical outcomes, including upon stem cell engraftment/function itself.

#### 2.3.4. Single donor or multi-donor?

The number of donors used to make an FMT product has been a further area of discussion. Most centers have favored using a single donor per FMT manufactured on the grounds of safety, as this facilitates easy ‘look back’ identification and potential removal of a specific donor or FMT sample donation should there be an apparent adverse event related to an FMT, such as FMT-related transmission of infection. Theoretically, multi-donor preparations may result in a more diverse microbiome ecosystem being transplanted, and pooling of donors may allow for compensation when levels of particular bacterial taxa or metabolites that are important for success of an FMT are low in a particular stool donation for an FMT. While several trials within the field of FMT and UC have used pooled donors, systematic review and meta-analysis of the evidence base appear to be contradictory with regards to whether this has any impact.^133,134^ In a different setting, a recent single arm study explored the impact of pooled donor fecal material in the treatment of steroid-resistant gastrointestinal graft-versus-host disease; pooled material was characterized by higher microbial richness and reduced batch-to-batch variability compared to single donors, and responding patients having a higher level of richness, correlating with engraftment of donor microbiome material from multiple donors.^131^

## 3. Future perspectives

The success of FMT in recurrent CDI – coupled with promising findings in a range of non-CDI settings – has kept levels of global enthusiasm high for the FMT as a springboard for development of a range of other ‘microbiome therapeutics’. However, given the variability of response in different studies (particularly non-CDI-related), there are a number of possible considerations or responses that might be made to account for this:

- *Overstated contribution of gut microbiome toward a particular disease*: The evidence supporting the contribution of the gut microbiome toward different conditions is at a very variable stage dependent upon the condition. While the evidence for a microbiome contribution toward CDI, IBD, and certain other conditions is unequivocal, this has for some conditions been based upon relatively small cross-sectional human studies with clear potential confounders, or based upon animal models of disease with debatable translational relevance. In such conditions, it may be therefore that the gut microbiome has only a minor or potentially trivial contribution toward such conditions, and it therefore being overambitious to expect that FMT would provide any meaningful benefit.
- *Ongoing uncertainty regarding optimal donor selection, donor–recipient matching, and procedural factors*: Many randomized FMT studies extrapolate optimal conditions for such factors based upon CDI studies, when clearly a much more nuanced range of considerations may be required for use of FMT in other conditions, as outlined in this review. A negative study of a trial of FMT in a non-CDI setting may therefore not necessarily reflect futility, but potentially that any of the variables discussed in this paper that are relevant for that disease state having not been considered. Based on available evidence, current FMT guidelines still to a large degree present best practice in, e.g. donor selection, based upon who to rule out (considering safety considerations and risk mitigation) rather than on positive biological or clinical characteristics.^13,14^ This is perhaps a reflection of our more general ongoing difficulty with defining a ‘healthy gut microbiome’.^135^

A further relevant point of discussion is what the ideal placebo/control should be in FMT clinical studies, and whether this may influence their outcome. Given the difficulty in making a mock FMT preparation, a number of studies have used autologous FMT (i.e. participant patients receiving back their own stool) as a control arm, but it is feasible that this may not entirely inert. Supportive of this, in a double blind RCT of FMT in treating recurrent CDI – where patients were randomized to receive healthy donor stool or autologous FMT – healthy donor stool was significantly more effectively than autologous FMT, but 62.5% of patients receiving autologous FMT (*n* = 15/24) still entered remission.^136^ In an era where capsulized FMT is increasingly used in FMT RCTs, it is obviously easier to manufacture a suitable placebo, and autologous FMT is no longer required so often.

Going forward, rather than viewing FMT as the perfect microbiome therapeutic, it is perhaps more reasonable to recognize its value as a discovery tool for us to further explore the contribution and impact of the gut microbiome in different human diseases, as part of a range of other experimental and investigative techniques. Evidence of clinical benefit in a human FMT study may inform mechanistic investigation, and itself inform future clinical studies. An example of this model of how human FMT studies may productively inform basic science – and vice versa – to better understand human disease and advance therapies is given in Figure 2, using FMT and MDRO as an example.

## 4. Conclusions

Over the past few years, significant progress has been made in understanding the applications of FMT in repairing the gut microbiome. Despite this, a large number of gaps remain regarding the contribution of clinical, biological, and procedural factors that may influence efficacy and outcome. The most productive advances to date are those where clinical and translational considerations are made in tandem, with a focus upon mechanistic understanding. This is a rapidly evolving field, and there is still much to be understood before the full potential of FMT can be realized in clinical practice.

## Supplementary Material

Supplementary Material Gut Microbes review.docx
