# Supplementary material for: Faecal (or intestinal) microbiota transplant: a tool for repairing the gut microbiome
Source: Gut Microbes. 2024 Nov 5;16(1):2423026. doi: 10.1080/19490976.2024.2423026 (PMC11540080; doi:10.1080/19490976.2024.2423026)
Supplement: Supplementary Material Gut Microbes review.docx [file KGMI_A_2423026_SM7647.docx]

**Supplementary Material:**

**Faecal (or Intestinal) Microbiota Transplant: A Tool for Repairing the Gut Microbiome**

1. **Summary of data related to prebiotics and probiotics related to certain communicable and non-communicable diseases:**

The beneficial effects of probiotics and prebiotics (as well as in combination) have been already explored in the context of a number of specific diseases states, both infectious as well as non-communicable.

For example, probiotic microorganisms (including lactic acid producing bacteria) - as well as prebiotics - were found to offer various health benefits attributed to their ability to modulate the immune system, reduce inflammation, and exhibit anti-cancer properties. Notably, a probiotic bacterial strain, *Lactobacillus casei* ATCC 393, has shown significant anti-proliferative effects in colon cancer, as demonstrated both *in vitro* using colon cancer cell lines and *in* vivo in animal models^1 2^. Additionally, probiotic therapies have been shown to reverse the detrimental influence of antibiotics on gut microbiota composition and function during cancer therapy, alleviating oxaliplatin-induced intestinal and liver damage in tumour-bearing mice^3^. Prebiotic treatment also holds great promise in cancer prevention and treatment^4^. The protective effects of short-chain fatty acids (SCFAs) against various diseases have been previously documented, showing their role in enhancing gut epithelial integrity, regulating metabolism, and boosting immune responses^4^.

Several clinical trials have explored the effects of probiotic and prebiotic use in the induction of remission in active ulcerative colitis (UC) and assessment of disease outcomes. Treatment of UC patients with *Bifidobacterium*-, *Lactobacillus*- and *Escherichia*-based probiotic strains (e.g., *B. infantis* 35624, *B. longum* 536, *E. coli* Nissle 1917, *L. casei*, *Lactobacillus delbruekii, L. fermentum, L. reuteri, L. rhamnosus*) as well as combination of those as part of commercially available probiotic supplements (VSL, BIO-THREE, Bifid Triple Viable), have shown to improve different metrics of UC activity^5^. This includes an improvement in activity-related scoring systems (including the UCDAI and SCCAI scores), improvement in stool frequency, and reduced colon mucosa inflammation (characterised by increase in IL-10 and IL-12p40, and a decrease in TLR-2 expression and IL-12p40 production)^5^. Enhanced clinical outcomes in UC were also previously-observed following the use of oligofructose-enriched inulin, inulin-type β-fructans, fructo-oligosaccharides, and galacto-oligosaccharides, as characterised by reduction in stool calprotectin, increase in clinical remission and alteration in immune-related genes expression^6^. Additionally, the role of *Lactobacillus*-based probiotics has been also found to prevent recurrent urinary tract infections secondary to vaginal colonization with *L. crispatus^7^.*

**References:**

1. Tiptiri-Kourpeti A, Spyridopoulou K, Santarmaki V, et al. Lactobacillus casei Exerts Anti-Proliferative Effects Accompanied by Apoptotic Cell Death and Up-Regulation of TRAIL in Colon Carcinoma Cells. *PLoS One* 2016;11(2):e0147960. doi: 10.1371/journal.pone.0147960 [published Online First: 20160205]

2. Irecta-Najera CA, Del Rosario Huizar-Lopez M, Casas-Solis J, et al. Protective Effect of Lactobacillus casei on DMH-Induced Colon Carcinogenesis in Mice. *Probiotics Antimicrob Proteins* 2017;9(2):163-71. doi: 10.1007/s12602-017-9253-2

3. Chang CW, Liu CY, Lee HC, et al. Lactobacillus casei Variety rhamnosus Probiotic Preventively Attenuates 5-Fluorouracil/Oxaliplatin-Induced Intestinal Injury in a Syngeneic Colorectal Cancer Model. *Front Microbiol* 2018;9:983. doi: 10.3389/fmicb.2018.00983 [published Online First: 20180515]

4. Delzenne NM, Bindels LB, Neyrinck AM, et al. The gut microbiome and dietary fibres: implications in obesity, cardiometabolic diseases and cancer. *Nat Rev Microbiol* 2024 doi: 10.1038/s41579-024-01108-z [published Online First: 20241010]

5. Huang C, Hao W, Wang X, et al. Probiotics for the treatment of ulcerative colitis: a review of experimental research from 2018 to 2022. *Front Microbiol* 2023;14:1211271. doi: 10.3389/fmicb.2023.1211271 [published Online First: 20230706]

6. Kennedy JM, De Silva A, Walton GE, et al. A review on the use of prebiotics in ulcerative colitis. *Trends Microbiol* 2024;32(5):507-15. doi: 10.1016/j.tim.2023.11.007 [published Online First: 20231207]

7. Stapleton AE, Au-Yeung M, Hooton TM, et al. Randomized, placebo-controlled phase 2 trial of a Lactobacillus crispatus probiotic given intravaginally for prevention of recurrent urinary tract infection. *Clin Infect Dis* 2011;52(10):1212-7. doi: 10.1093/cid/cir183 [published Online First: 20110414]
